# LATE CONSERVATIVE TREATMENT FOR ESOPHAGEAL PERFORATION BY FOREIGN BODY

**DOI:** 10.1590/0102-672020180001e1467

**Published:** 2019-12-20

**Authors:** Victor Alexander Fialho ROCHA, Wesley da Costa REIS, Kévin da Silva SOUZA, Reyner Abrantes STIVAL, Cristiano de Magalhães NUNES, Wellington José dos SANTOS

**Affiliations:** 1 Pontifícia Universidade Católica de Goiás, Goiânia, GO, Brasil; 2 Hospital de Urgências de Goiánia, Goiânia, GO, Brazil.

**Keywords:** Foreign bodies, Endoscopy, gastrointestinal, Esophageal perforation, Corpos estranhos, Endoscopia gastrointestinal, Perfuração esofágica

## INTRODUCTION

The management of the traumatic perforation of the esophagus constitutes challenging situation, since that is unusual condition; the diagnosis is hindered by the nonspecific or discrete symptomatology and the treatment standardization is also hindered by the variety of the causes and its consequences^3^ ^,^ ^4^ ^,^ ^6^ ^,^ ^10^.

Among the causes, the foreign body constitutes about 10% of the cases and the esophageal portion more commonly injured is the thoracic, followed by the cervical and abdominal^7^.

The diagnosis can be firmed by the association of the clinical manifestation and the evaluation by radiological examinations^5^ ^,^ ^9^ ^,^ ^10^. The high digestive endoscopy can be also used for diagnosis and treatment; however, the delay is associated with the higher morbidity and mortality^7^. Furthermore, because of the individual character of the behavior therapeutic, the choice of a conservative treatment rather than surgical approach is still controversial^4^.

This current report has the aim to identify atypical outcome and to raise alternative conditions for the good management of the esophageal perforation. This report was approved by the Ethics Committee of the Hospital de Urgências de Goiânia, GO, Brazil. 

## CASE REPORT

**FIGURE 1:**
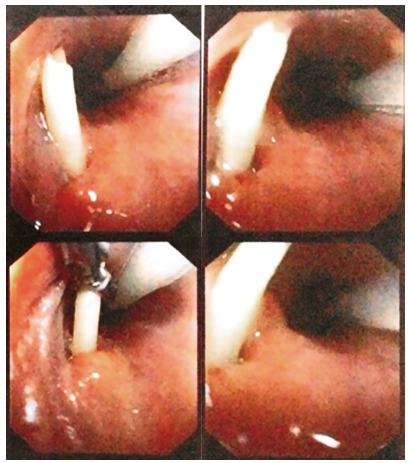
High digestive videoendoscopy showing fragment of filiform bone in left lateral esophageal wall and nasogastric tube in the right lateral wall

A 25-year-old-male was admitted in the Emergency of the Hospital de Urgências de Goiânia with the report of intake of foreign body and its impaction in the upper esophagus with five days of evolution. He was submitted to the higher digestive endoscopy on his hometown with frustrated attempt to remove in the same day of admission. He was hemodynamically stable and afebrile in the moment of his admission, having a normal arterial pressure, having 72 bpm and Sat0_2_ 94%. At the hospital, it was done high digestive videoendoscopy, where it was evidenced the presence of foreign body perforating the upper cervical esophagus (Figure 1).

**FIGURE 2:**
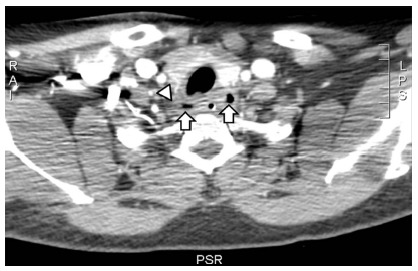
Cervical computed tomography (axial section, with contrasts, venous phase) emphasizing emphysema (long arrows) and perilesional inflammatory process (head of arrow)

The foreign body was removed using endoscopic device and, soon after, the patient was submitted to the computed tomography, showing emphysema and perilesional inflammatory process, but absence of collections (Figure 2). The hemogram showed relative and absolute eosinophilia of 18% and 1170/mm^3^ (reference values: 1-4%, 45-400/mm^3^), respectively. It was also done thoracic radiography with no alterations.

The conservative treatment was chosen with nasogastric intubation by endoscopy and antibiotic therapy (intravenous ciprofloxacin 400 mg each 12 h and intravenous metronidazole 500 mg each 8 h) during 10 days and semi-intensive monitoring. It was installed also cervical drain that drained clear liquid and without blood. He had good evolution and was discharged from hospital after 15 days of hospitalization. A month after he was in outpatient care without symptoms or complications

## DISCUSSION

It is known that in cases in which the diagnosis and the treatment are started after 24 h from the esophageal injury occurrence - in other words, delayed -, greater complications are related demanding aggressive operations with higher morbimortality^5^ ^,^ ^7^ ^,^ ^9^. 

The diagnostic confirmation of the esophageal perforation can be obtained by the high digestive endoscopy, that can be also used for the purpose therapeutics and by radiological exams with contrasts, as the computed tomography looking for emphysema and perilesional inflammatory process. Impacted sharp objects in the cervical esophagus must be removed by high digestive endoscopy, with success rate in 95%^8^.

Diagnostic done, the treatment is: infection control, nutrition maintenance and injured digestive tract repair with reinforcement suture. However, the procedure for treatment in cases with longer days is removal of the foreign body, no injury suturing, antibiotic therapy and enteral nutrition, similar to this report^2^.

In a Brazilian public hospital^7^ the mortality was smaller in cervical perforation than thoracic and abdominal, as well as statistically significant smaller in patients that received surgical treatment. However, if the esophageal perforation be buffered, with no evidence of sepsis or communication with the pleural or peritoneal cavity, it is recommended fasting, hydration, preferably enteral nutrition support and antibiotic therapy during 14 days^11^.

The surgery is suitable in cases with wide perisophageal injury, associated to the clinical condition that suggests sepsis, pneumothorax, mediastinal emphysema and respiratory failure. In patients with stable and small injuries there is no demand for immediate surgical repair, but it is recommended intensive monitoring and follow-up by experienced surgeon and radiological exams^1^ ^,^ ^8^ ^,^ ^10^ ^,^ ^11^.
